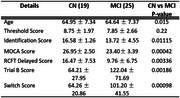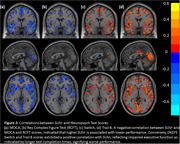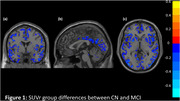# Relationship between Aβ PET Imaging and Cognitive Test Scores in MCI

**DOI:** 10.1002/alz70856_103885

**Published:** 2025-12-26

**Authors:** Senal Peiris, Qing Yang, Anupa Manjitha Ekanayake Mudiyanselage, Rommy Elyan, Katie Geesey, Sangam Kanekar, Jens Will, Paul Eslinger, Prasanna Karunanayaka

**Affiliations:** ^1^ Pennsylvania State University College of Medicine, Hershey, PA, USA

## Abstract

**Background:**

Amyloid imaging with positron emission tomography (PET) is important for the diagnosis and treatment of Alzheimer's disease (AD). Probable AD is typically diagnosed with magnetic resonance imaging (MRI) by identifying anatomical changes unique to AD. Due to natural variations in brain volume, structural MR imaging is rather ambiguous. The standardized uptake value ratio (SUVr), derived from PET imaging, is a more accurate quantitative measure for accurate diagnosis of AD. Furthermore, cognitive decline and olfactory impairment are common preclinical symptoms of AD. To better understand brain‐behavior relationships in AD spectrum, here we explored relationships with SUVr and behavioral (neuropsychological/olfactory) test scores. We hypothesized that there will be negative correlations between neuropsychological test scores and SUVR values.

**Method:**

We analyzed Amyloid beta PET (Aβ) scans from 44 subjects (19 CN, 25 MCI). PET scans were conducted on a Siemens Biograph mCT 20 scanner with 10‐minute scans taken 30 minutes post‐injection. The images were pre‐processed and registered to T1 MRI. Standardized Uptake Value Ratio (SUVr) was calculated with Clinica software. Correlation analyses was performed with DPABI software.

**Result:**

Significant group differences in neuropsychological scores were observed between CN and MCI (Figure 1), were observed between CN and MCI figure. Similarly, SUVr differences were observed between CN and MCI (Figure 2). The impact of SUVr on various cognitive domains in terms of correlation analyses were investigated and shown in Figure 3. All correlations yielded highly significant results at *p* <0.005.

**Conclusion:**

This study provides valuable insights into the relationship between Aβ PET imaging and cognitive function in both the (CN) and (MCI) subjects. The correlation between SUVr and olfactory function was insignificant. Olfactory measures such as Identification and Threshold may require further improvement to enhance their utility in AD pathophysiology. Overall, our findings show that higher amyloid beta deposition is linked to poorer cognitive performance in MCI. Our results may offer potential avenues to streamline the Neuropsychological test battery for AD by closely tying most salient metrics to amyloid beta deposition.